# A Broadband Photodetector Based on Non-Layered MnS/WSe_2_ Type-I Heterojunctions with Ultrahigh Photoresponsivity and Fast Photoresponse

**DOI:** 10.3390/ma17071590

**Published:** 2024-03-30

**Authors:** Chaojie Xie, Yibin Yang, Kunle Li, Xuanhao Cao, Shanshan Chen, Yu Zhao

**Affiliations:** Guangdong Provincial Key Laboratory of Information Photonics Technology, Guangdong Provincial Key Laboratory of Functional Soft Condensed Matter, School of Material and Energy, Guangdong University of Technology, Guangzhou 510006, China; 2112102193@mail2.gdut.edu.cn (C.X.); yangyibin@gdut.edu.cn (Y.Y.); 1112302011@mail2.gdut.edu.cn (K.L.); cxh2903@gmail.com (X.C.)

**Keywords:** MnS, broadband photodetectors, type-I heterojunctions

## Abstract

The separation of photogenerated electron–hole pairs is crucial for the construction of high-performance and wide-band responsive photodetectors. The type-I heterojunction as a photodetector is seldomly studied due to its limited separation of the carriers and narrow optical response. In this work, we demonstrated that the high performance of type-I heterojunction as a broadband photodetector can be obtained by rational design of the band alignment and proper modulation from external electric field. The heterojunction device is fabricated by vertical stacking of non-layered MnS and WSe_2_ flakes. Its type-I band structure is confirmed by the first-principles calculations. The MnS/WSe_2_ heterojunction presents a wide optical detecting range spanning from 365 nm to 1550 nm. It exhibits the characteristics of bidirectional transportation, a current on/off ratio over 10^3^, and an excellent photoresponsivity of 108 A W^−1^ in the visible range. Furthermore, the response time of the device is 19 ms (rise time) and 10 ms (fall time), which is much faster than that of its constituents MnS and WSe_2_. The facilitation of carrier accumulation caused by the interfacial band bending is thought to be critical to the photoresponse performance of the heterojunction. In addition, the device can operate in self-powered mode, indicating a photovoltaic effect.

## 1. Introduction

In recent years, ultra-thin two-dimensional (2D) nanomaterials have attracted extensive attention due to their unconventional physical and chemical properties [1,2]. In particular, 2D material heterojunctions with atomically sharp heterointerfaces, adjustable energy band alignments, and attractive interlayer couplings [3,4,5] have emerged as important components in nanoelectronic and optoelectronic devices [6,7,8,9,10]. In particular, the band alignments of 2D heterojunctions affect the transport of photogenerated carriers, which is crucial for the construction of high-performance and wide-band responsive photodetectors. Most photodetectors are based on the building block of heterojunctions possessing type-II band alignment on account of the efficient separation of electron–hole pairs generated in either component [11,12,13]. In this band structure, the conduction band minimum (CBM) and valence band maximum (VBM) of a type-II heterojunction are separately located in its two different components, whereas, for a type-I heterojunction, both the CBM and VBM are located in the narrow bandgap side, which becomes the potential well for photogenerated carriers. In this case, either electrons or holes generated in the barrier side can be injected into the well side through the heterointerface, and the opposite carriers are collected on the electrode of barrier side, while carriers generated in the well side cannot be effectively separated due to the energy barriers at the interface. Therefore, photodetectors based on a type-I heterojunction show spectral response selectively decided by the barrier component, which usually corresponds to ultraviolet (UV) or visible range. The effective separation of photogenerated electron–hole pairs in type-I band alignment is proved to be very helpful to realize ultrafast and sensitive photodetection [14]. However, taking full advantage of improving carrier collection efficiency, the broadband response based on a type-I heterojunction is still a challenge.

Fortunately, properly designed heterojunctions can break through the limitations of the material itself to work in UV to visible or even near-infrared band [15]. The conduction and valence band offsets as well as the band bending are determined by the properties of components, and can be synchronously modulated by electric field. Therefore, rational designs of the type-I band alignment and carrier transport process with electric field modulation are crucial for constructing novel photodetectors based on type-I heterojunctions with broadband response and efficient carrier separation. Compared with type-II heterojunctions, which are widely studied and fabricated, type-I heterojunctions are less studied, and the optical response principle and regulation mechanism are not fully understood. Hence, it is highly desirable to design and fabricate a novel type-I heterojunction, analyze its tunable band alignment modulated by electric field, and investigate its optoelectronic performance as a photodetector.

The emergence of 2D non-layered materials with three-dimensional chemically bonded crystal structures not only greatly extends the scope of the inherent layered 2D materials, but also demonstrates a range of interesting properties due to the large number of unsaturated dangling bonds on the surface. These surface active sites make them ideal materials for surface active applications such as catalysts [16], supercapacitors [17], and photodetectors [18,19]. As an important member of the group of non-layered materials, MnS, the group VIIB transition metal chalcogenide, exhibits excellent electronic, photoelectric, and magnetic properties [20,21]. The self-containing manganese vacancy in α-MnS (stable rock-salt-type structure) acts as a receptor, resulting in p-type conductive behavior with a wide band gap of 2.7 eV [22,23]. The synthesized α-MnS crystals show excellent photoresponsivity, environmental stability, and flexibility, which indicates that MnS has considerable application potential in flexible electronic and optoelectronic devices [23]. The wide band gap of MnS has benefits in its application in the UV photoresponse [24]. In order to construct the type-I heterojunction based on MnS with a broadband photoresponse, a narrow band gap material is needed as the well layer. WSe_2_ has an indirect band gap of 0.9–1.6 eV, and the heterojunction detector based on WSe_2_ shows a broad spectral response ranging from visible and near-infrared light. In addition, the WSe_2_ field-effect transistor exhibits high carrier mobility (70.1 cm^2^V^−1^s^−1^) and ON/OFF current ratio (over 10^6^) [25,26,27]. Accordingly, heterojunctions formed by stacking WSe_2_ and other 2D materials will help to broaden the response band and improve the photoresponse performance.

In this work, non-layered MnS grown by chemical vapor deposition (CVD) and mechanically exfoliated WSe_2_ were used to construct the 2D heterojunction. Type-I band alignment of the MnS/WSe_2_ heterojunction was verified by first-principles calculations. The MnS/WSe_2_ heterojunction exhibits excellent photoresponsive properties and tunable band alignment under electric field modulation. The device also shows the characteristic of bidirectional transportation. The influence of carrier accumulation and depletion state at the interface on the photoresponse performance of the device under the two bias cases is discussed. The photodetector based on MnS/WSe_2_ possesses a broadband detection, a high photoresponsivity, and a fast response speed in the visible light range, which is better than the performance of a single component material (WSe_2_ or MnS). Furthermore, the photovoltaic effect of the device has been evaluated.

## 2. Materials and Methods

### 2.1. Synthesis of MnS Nanosheets

The mica substrate was ultrasonically treated in ethanol, acetone, and isopropyl alcohol for 15 min to remove organic pollutants before the preparation of MnS nanosheets. The whole preparation process was executed in a two-temperature zone CVD system. The precursors were S powder (99.98%, Alfa, Shanghai, China) and the mixed powder of high purity MnCl_2_ powder (99.999%, Alfa, Shanghai, China) with some NaCl. S powder and the mixed powder were separately placed in two quartz boats in the middle of the high temperature region and low temperature zone of the furnace, respectively. The temperature in the high temperature region was maintained at 640~660 °C, and the low temperature region was maintained at 180 °C. The mica substrate was placed on a quartz sheet about 5 cm from the MnCl_2_/NaCl powder at the downstream end of the high temperature zone. Before heating, 200 sccm Ar (96%) and H_2_ (4%) mixture was injected into the tube for 30 min to remove O_2_, which ensured a stable reaction environment. Then, heating the furnace to the required growth temperature, a MnS nanosheet was grown for 5–10 min with a continuous flow of 20–30 sccm under normal pressure. Finally, the heating was stopped, and the flow rate was increased to 100 sccm. The mica substrate was naturally cooled to normal temperature.

### 2.2. Preparation of WSe_2_ Nanosheets

WSe_2_ nanosheets were mechanical exfoliated from the commercial bulk WSe_2_ (ONWAY, Shanghai, China) on blue tape (NITTO, Hongkong, China) and transferred to the Si/SiO_2_ substrate.

### 2.3. Characterizations

Optical microscope (Motic, Xiamen, China) was used to characterize the morphology. Material composition was characterized by X-ray photoelectron spectroscopy (XPS) (Thermo Fisher, Waltham, MA, USA). X-ray diffraction (XRD) (Bruker, Bilerika, MA, USA) and high-resolution transmission electron microscopy (HRTEM) (FEI, Hillsboro, OR, USA) were applied to characterize microstructure. The electric and photoelectric properties were studied on a four-probe table (SEMISHARE, Shenzhen, China) combined with the 2636B source meter (KEITHLEY, Cleveland, OH, USA). For this, 365, 405, 532, 808, and 1550 nm lasers were applied as the probing light sources.

## 3. Results and Discussion

**Figure 1:**
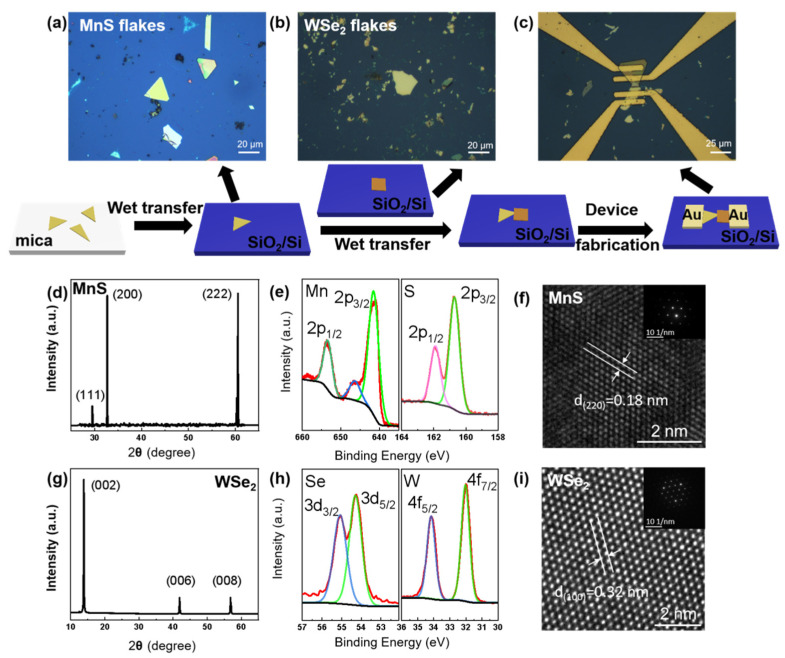
(**a**–**c**) The schematic diagram of the preparation process of the MnS/WSe_2_ heterojunction. The MnS grown by CVD and WSe_2_ removed by blue tape were used to form a vertical stack through wet transfer process. Then the device fabrication was completed by the electron beam evaporation. (**d**,**g**) XRD patterns, (**e**,**h**) XPS patterns (The black line is the baseline. The red lines are the raw data and the remaining lines of different colors are the fitted lines of the XPS feature peaks), and (**f**,**i**) HRTEM images with the illustration of selected area electron diffraction (SAED) patterns of MnS and WSe_2_ nanosheets, respectively.

Two-dimensional MnS flakes were grown on mica substrates by the CVD method and then were transferred onto the SiO_2_ (300 nm)/Si substrate through the wet transfer process. WSe_2_ was removed by a mechanical exfoliation method and transferred onto the above SiO_2_/Si substrate. Then, MnS and WSe_2_ were used to constitute a vertical stack junction through the wet transfer process. After that, 50 nm Au was used to cover the MnS/WSe_2_ heterojunction by a standard lithography process and electron beam evaporation. The above preparation method is shown in Figure 1a–c and the detailed preparation process is in the e.g., Section 2.

The constituent materials and microstructure of the heterojunction are characterized by XRD, XPS, and HRTEM. The formation of pure MnS crystals and their single-crystal nature are confirmed by the XRD result, as shown in Figure 1d. The XRD peaks appear at positions of 2θ = 29.5°, 32.7°, and 60.5°, corresponding to the (111), (200), and (222) planes of MnS, which is well-conformed with the criterion MnS pattern (PDF no. 06-0518). In Figure 1e, the XPS spectrum exhibits that peak positions at 653.5 eV and 641.4 eV belong to Mn 2p_1/2_ and Mn 2p_3/2_, respectively. Those at 161.9 eV and 160.7 eV are designated as S 2p_1/2_ and S 2p_3/2_ [28]. As shown in Figure 1f, the HRTEM image of MnS exhibits a (220) crystal plane with a spacing of 0.18 nm, matching the description with the XRD result. In the inset of Figure 1f, the SAED pattern exhibits one set of hexagonal diffraction spots, indicating the high quality of the MnS single crystal. As shown in Figure 1g, the major XRD peaks appear at positions of 2θ = 13.8°, 41.8°, and 56.8°, corresponding to the (002), (006), and (008) planes of WSe_2_, which is well-matched to the standard WSe_2_ pattern (PDF no. 38-1388). The peaks at 34.1 eV and 32.0 eV in Figure 1h are assigned to W 4f_5/2_ and W 4f_7/2_, respectively, while the other two peaks at 55.1 eV and 54.3 eV are designated as Se 3d_3/2_ and Se 3d_5/2_ states of WSe_2_ [29], respectively. As shown in Figure 1i, the spacing of WSe_2_ is 0.32 nm in the HRTEM image, corresponding to the WSe_2_ (100) plane. The SAED pattern in the inset of Figure 1i shows one set of hexagonal diffraction spots, indicating the high quality of the WSe_2_ single crystal.

**Figure 2:**
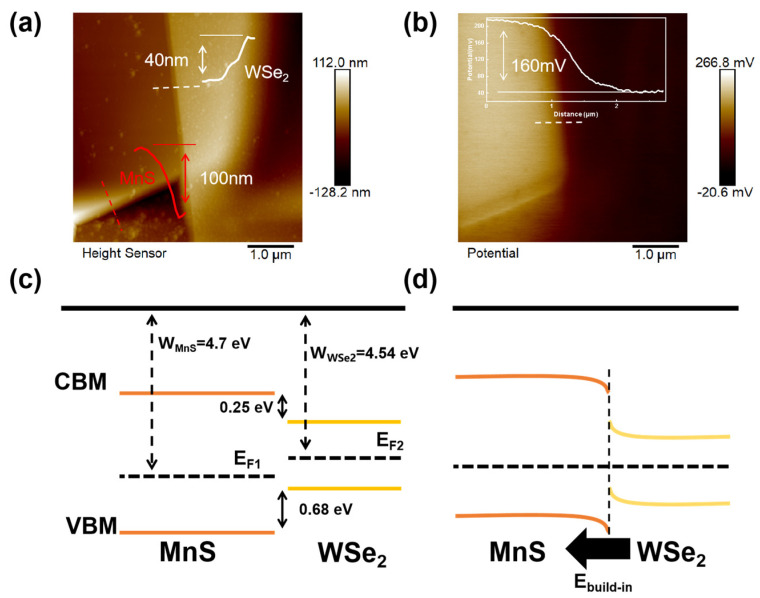
(**a**) AFM image of MnS/WSe_2_ interface with insets of its height profile. (**b**) The difference of potential between MnS and WSe_2_ with an inset of the potential height profile. Diagrams of the energy band alignments between MnS and WSe_2_ (**c**) before and (**d**) after contact.

As shown in Figure 2a, the thicknesses of the MnS and WSe_2_ nanosheets are studied by AFM and calculated to be 100 and 40 nm, respectively. The conductivity type of MnS is experimentally measured as p-type, shown in Supplementary Figure S1, and WSe_2_ is measured as bipolar conductivity [30]. Then, as shown in Figure 2b, the built-in contact potential difference at the interface between MnS and WSe_2_ is obtained by Kelvin probe force microscopy (KPFM) measurement. To illustrate the surface potential distribution (SPD) of MnS and WSe_2_ nanosheets with respect to the tip region of AFM, the following equations can be expressed:

(1) $$\begin{array}{c} e{SDP}_{MnS}=W_{tip}-W_{MnS} \end{array}$$

(2) $$e{SDP}_{{WSe}_{2}}=W_{tip}-W_{{WSe}_{2}}$$

where e is the electron charge, and $W_{tip}$, $W_{MnS}$, and $W_{{WSe}_{2}}$ are the work functions of the AFM tip, MnS, and WSe_2_ flakes, respectively. In order to obtain the Fermi energy level difference ΔE_F_ between MnS and WSe_2_, the following equation is expressed:

(3) $$∆E_{F}=W_{MnS}-W_{{WSe}_{2}}=e{SDP}_{MnS}-e{SDP}_{{WSe}_{2}}$$

As shown in Figure 2c, the work function difference between MnS and WSe_2_ is about 0.16 eV. $W_{MnS}$ and $W_{{WSe}_{2}}$ are about 4.7 and 4.54 eV, respectively, by calculation through $W_{tip}$ as 4.5 eV. The band diagrams of MnS and WSe_2_ are obtained by the first-principles calculations. The calculations are performed using the projector-augmented plane-wave (PAW) method within the work of density functional theory (DFT) in the VASP software package (VASP 5.4). The generalized gradient approximation (GGA) of Perdew, Burke, and Ernzerhof (PBE) function is employed for the electron exchange and correlation [31]. A vacuum of about 20 Å is applied to eliminate the interaction between adjacent images. The cutoff energy of the plane-wave basis set is 450 eV. The first Brillouin zone is sampled with a (15 × 15 × 1) Monkhorst−Pack grid for the relaxation of MnS and WSe_2_. All of the structures are fully relaxed with a force tolerance of 0.02 eV/Å. In addition, 1 × 1 supercells with 1~4 layers of both MnS and WSe_2_ are constructed, as shown in Supplementary Figure S2a–e. The average band offsets of CBM and VBM between MnS and WSe_2_ are 0.25 eV and 0.68 eV, respectively, and the band alignments of MnS and WSe_2_ before contact with ΔE_F_ of 0.16 eV is shown in Figure 2c. Finally, taking Fermi energy level difference into consideration, the MnS/WSe_2_ heterojunction still shows a type-I band structure with a built-in electric field from WSe_2_ to MnS, as shown in Figure 2d.

**Figure 3:**
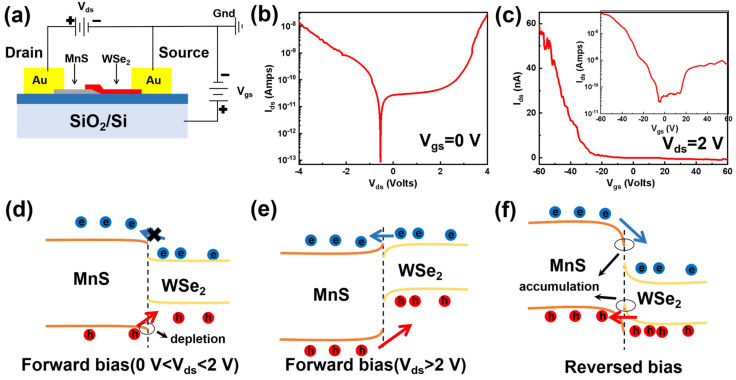
(**a**) The schematic diagram for electrical measurements. (**b**) The I_ds_–V_ds_ (I_ds_ and V_ds_ is the current and voltage between the source and drain, respectively) curve of the MnS/WSe_2_ heterojunction at V_gs_ = 0 V. (**c**) The transfer (I_ds_–V_gs_) curve of MnS/WSe_2_ heterojunction at V_ds_ = 2 V, inset is the identical curve in logarithmic plot. Energy band diagrams of the MnS/WSe_2_ heterojunction under (**d**) forward bias (0 V < V_ds_ < 2 V), (**e**) forward bias (V_ds_ > 2 V), (**f**) reversed bias.

Applied bias on the MnS/WSe_2_ field-effect transistor (FET) is demonstrated in Figure 3a, with MnS as source and WSe_2_ as the drain electrodes. To further investigate the electrical characteristics of the device as shown in Figure 3b,c, the analysis combined with band alignments is conducted as shown in Figure 3d–f. The tunable band alignment and carrier transport process under electric field modulation can be divided into the following three processes described below.

When forward bias (0 V < V_ds_ < 2 V) is applied, electrons in the WSe_2_ conduction band cannot normally move towards the drain electrode due to the high potential barrier of MnS, while holes in the MnS valence band can move towards the source electrode. The band bending at the interface forms a hole depletion region on the MnS side and an electron depletion region on the WSe_2_ side, which hinders carrier transport in forward bias. These explain the low dark current under 0–2 V bias in the I_ds_–V_ds_ curve, as shown in Figure 3b.

When V_ds_ is greater than 2 V, the current increases rapidly as the forward bias continues to increase because the electron barrier no longer exists, due to the energy band upward shift of WSe_2_. Therefore, the electrons in WSe_2_ can transfer to the drain electrode and the holes in the MnS valence band can move towards the source electrode.

Under reverse bias, with the energy band of WSe_2_ moving down, electrons in the MnS conduction band can move easily toward the source electrode, and the confinement effect of holes in the WSe_2_ valence band gradually weakens until the VBM of WSe_2_ is lower than of the VBM of MnS. Consequently, holes in the WSe_2_ valence band can transfer toward the drain electrode, and the heterojunction presents a state of reverse conduction. This explains the I_ds_–V_ds_ curve where the current increases when the voltage of V_ds_ is less than 0 V.

The I_ds_–V_ds_ curve in Figure 3b shows the bidirectional transport characteristics of the MnS/WSe_2_ heterojunction, and the reverse current is greater than the forward current at ±2 V. The voltage direction coinciding with the internal electric field increases the interface band bending, which results in the strengthened accumulation regions of electrons on the MnS side and holes on the WSe_2_ side. The situation better matches and facilitates carrier transport under reverse bias. The ambipolar behavior of the heterojunction is confirmed by transfer characteristic curve from Figure 3c. However, p-type characteristics dominate, explaining that the electrical conductivity of the device is mainly governed by the MnS channel. It can be seen from Figure 3c that the current on/off ratio of the device exceeds 10^3^.

**Figure 4:**
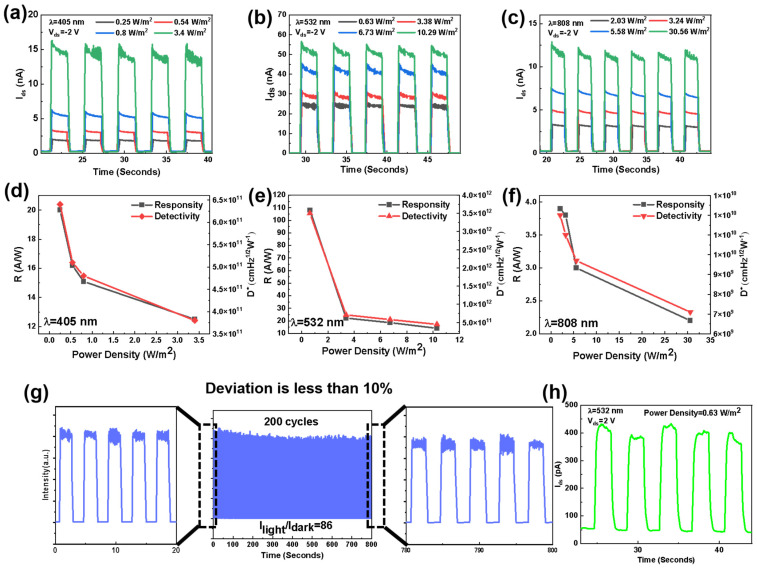
The photo current of the MnS/WSe_2_ heterojunction device (V_ds_ = −2 V and V_gs_ = 0 V) under probing light of (**a**) 405 nm, (**b**) 532 nm, and (**c**) 808 nm lasers with different optical power densities. (**d**–**f**) The corresponding responsivity and detectivity under irradiation lasers with corresponding wavelength. (**g**) The light current of the MnS/WSe_2_ heterojunction for 200 continuous cycles of irradiation (532 nm laser) (I_light_/I_dark_ is the ratio of dark current to photocurrent). (**h**) The photoresponse of the MnS/WSe_2_ heterojunction at V_ds_ = 2 V and V_gs_ = 0 V under the irradiation of 532 nm laser with 0.63 W/m^2^ power intensities.

The photoresponse performance of the heterojunction device is systematically investigated. As shown in Figure 4a–c, the photocurrent of the device is measured at V_ds_ = −2 V and under the probing light of 405, 532, and 808 nm lasers, respectively. The three parameters that evaluate the optical response performance of a device are responsivity (R_λ_), detectivity (D*), and external quantum efficiency (EQE). The photocurrent generated per unit power of the incident light per unit area of a photoelectric device is the responsivity R_λ_. The equation is expressed as follows

(4) $$R_{\lambda }=\frac{I_{ph}}{P_{\lambda }A}=\frac{I_{light}-I_{dark}}{P_{\lambda }A}$$

where I_light_ and I_dark_ are the photocurrents in light and dark conditions, respectively, and P_λ_ is illumination power density. The effectively irradiated area of the device is A. D* determines the capacity to detect weak optical signals [32,33,34] and is expressed as follows

(5) $$D^{*}=\frac{R_{\lambda }\sqrt{A}}{S_{n}}$$

where e is the charge of electron and S_n_ is the noise spectral density. The number of electron–hole pairs excited by one incident photon is defined as EQE. The expression is as follows

(6) $$EQE=\frac{hcR_{\lambda }}{e\lambda }$$

where h is Planck constant and λ is the incident wavelength. As shown in Figure 4d–f, the responsivity and detectivity under different optical power densities of 435, 532, and 808 nm lasers are calculated. As shown in Figure 4b,e, under the 532 nm laser with a power density of 0.63 W m^−2^, the photocurrent is 2.56 × 10^8^ A and the dark current is 2.17 × 10^10^ A. Fourier transform of the dark current traces gives the noise spectral density (S_n_) as a function of frequency, as shown in Figure S4. Considering the fast speed of the device, S_n_ can be extracted at a frequency of 20 Hz. The calculated maximal R_λ_, D*, and EQE are 108 A W^−1^, 3.5 × 10^12^ jones, and 25,100%, respectively. To comprehend the relationship between optical power density and optical response, the power-law I_ph_ ∝ P^α^ (α is the power index) is used to define the photocurrent. The fitted function relationship between photocurrent and optical power density is I_ph_ (A) ∝ 2.5 × 10^8^ [P(W/m^2^)]^0.79^. Furthermore, in order to study the stability, the device was irradiated for 200 consecutive cycles under a 532 nm laser. The optical switching of the device exhibits a steady response and only a slight attenuation of the photocurrent (deviation less than 10%) in Figure 4g. These results indicate that the MnS/WSe_2_ heterojunction has the potential for high-performance photoelectric detection.

The photoresponse performance of the heterojunction device measured at V_ds_ = 2 V and under the irradiation of 532 nm laser is also investigated, as shown in Figure 4h. The maximal R_λ_ is 0.34 A W^−1^ under forward bias, which is lower than that under reverse bias. It reveals the influence of carrier accumulation and depletion state at the interface on the photoresponse performance of the device.

**Figure 5:**
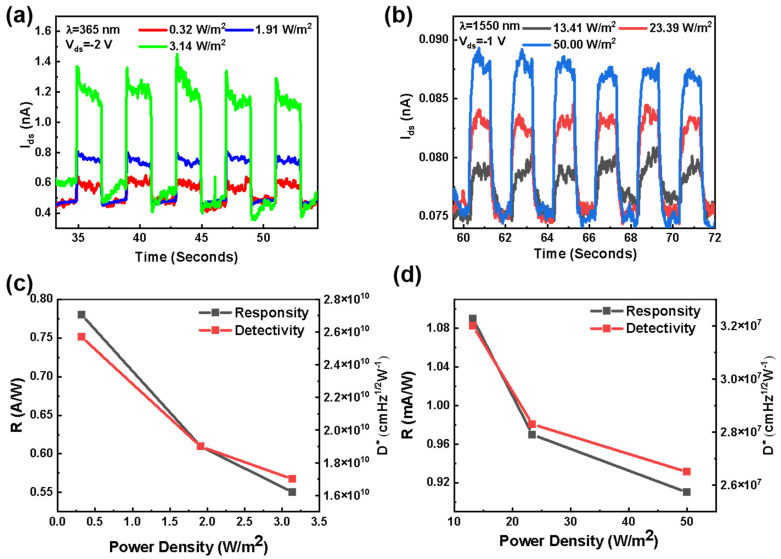
The photocurrent of the MnS/WSe_2_ heterojunction at V_ds_ = −2 V and V_gs_ = 0 V under (**a**) 365 nm laser and at V_ds_ = −1 V and V_gs_ = 0 V under (**b**) 1550 nm laser, with different optical power densities. The responsivity and detectivity under the irradiation of (**c**) 365 nm and (**d**) 1550 nm laser, respectively.

Except for the visible region, the light response for UV and near-infrared is studied subsequently, as shown in Figure 5a–d. When the 365 nm laser with a power density of 0.32 W m^−2^ illuminates the device, three parameters (R_λ_, D*, and EQE) are calculated to 0.78 A W^−1^, 2.5 × 10^10^ jones, and 260%, respectively. The heterojunction also generates a photocurrent under a 1550 nm laser with 13.41 W m^−2^. By calculation, R_λ_, D*, and EQE are 1.09 mA W^−1^, 3.2 × 10^7^ jones, and 7.9%. The resulting photocurrent is relatively low. The downward pulling of the WSe_2_ side band diagram forms a type-II band alignment under the reverse bias, as shown in Figure S3, which allows the photogenerated holes in WSe_2_ to transport directly into the VBM of MnS under the 1550 nm laser.

**Figure 6:**
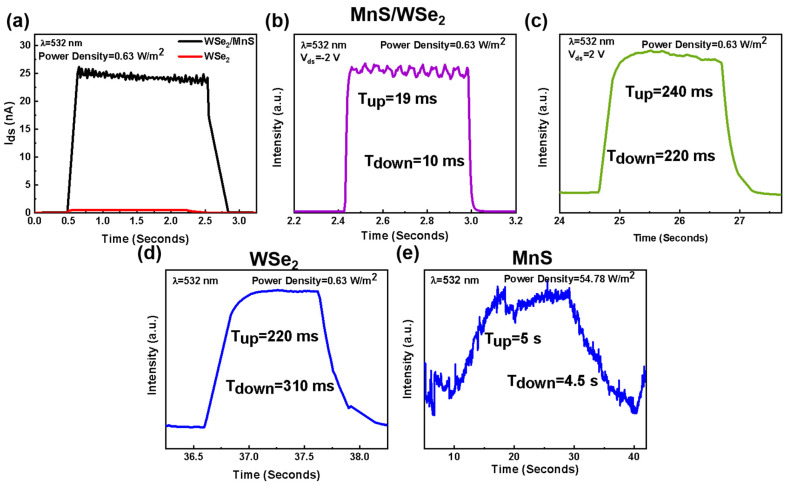
(**a**) The responding photocurrent of the MnS/WSe_2_ heterojunction and its constituent WSe_2_. The responding time of photocurrent of MnS/WSe_2_ heterojunction (**b**) at −2 V and (**c**) at 2 V, (**d**) WSe_2_ and (**e**) MnS.

To compare the properties of the heterojunction with its components, the photocurrents from the MnS/WSe_2_ heterojunction and individual materials of the same device are measured. The photocurrent of MnS/WSe_2_ heterojunction is nearly 50 times higher than WSe_2_ in Figure 6a, indicating that the separation of photogenerated electron–hole pairs in the type-I heterostructure effectively improves the optical responsivity. Meanwhile, the response time of the device, MnS, and WSe_2_ are measured (rising time T_up_ and falling time T_down_ are defined as the interval between the response going up from 10% to 90% and down from 90% to 10%, respectively) in Figure 6b–d. The rising time is 19 ms and falling time is 10 ms, which are two and one order of magnitude faster than MnS (5 s/4.5 s) and WSe_2_ (220 ms/310 ms), respectively. The band offset leads to photo-generated charge carrier (electron–hole pairs generated by light) separation at the heterojunction interface, thereby accelerating the transport speed of the charge carriers, and the response rate of the heterojunction is faster than that of the simple substance. Even after the 200 cycles shown in Figure 4g, there is almost no change in response time of the heterojunction. The response time of the heterojunction device at V_ds_ = 2 V, is 240 ms (rise time) and 220 ms (fall time), as shown in Figure 6c. It can be seen that, although the carrier separation efficiency of the heterojunction under forward bias is not as good as that under reverse bias, the heterojunction still generates an effective carrier separation with the forward bias modulation. Some key performances of reported broadband photodetectors are collected in Table S2, and it can be seen that the MnS/WSe_2_ heterojunction has great application potential.

**Figure 7:**
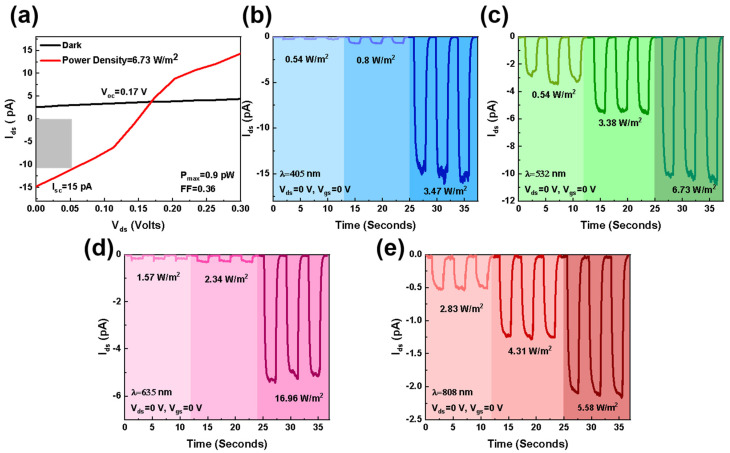
(**a**) I_ds_–V_ds_ curve at zero bias, under dark, and 532 nm laser irradiation. The MnS/WSe_2_ heterojunction is irradiated under (**b**) 405 nm, (**c**) 532 nm, (**d**) 635 nm, and (**e**) 808 nm lasers, and the photocurrent response at zero bias with different light power densities.

The photovoltaic performance of the device is researched under the zero-bias state. As shown in Figure 7a, when between bias and illuminated, the heterojunction exhibits obvious light response at zero bias, illuminated by a 532 nm laser with the power density of 6.73 W m^−2^. The short-circuit current (I_sc_) and open-circuit voltage (V_oc_) in Figure 7a are 15 pA and 0.17 V, respectively. By calculating the corresponding power of 0–0.17 V, it is concluded that P is the maximum when V = 0.053 V, which is the gray area in the figure (P_max_ = 0.9 pW). The fill factor (FF) is the important parameter for assessing the photovoltaic performance. It can be calculated as follows

(7) $$FF=\frac{P_{max}}{V_{oc}I_{sc}}$$

which is calculated to be 0.36. Figure 7b–e shows the device-generated photocurrents under irradiation of 405, 532, 635, and 808 nm lasers with different powers. This indicates the device works as a self-powered photodetector. The strongest light response is generated when the laser is 532 nm with 0.54 W m^−2^, and the corresponding photo and dark current are 3 pA and 0.1 pA, respectively. R_λ_, D*, and EQE are calculated as 9.7 mA W^−1^, 1.5 × 10^7^ jones, and 22.5%, respectively. It proves that the MnS/WSe_2_ heterojunction is a well-performing, self-powered photodetector and photovoltaic device. Under the zero-bias state, the wide-bandgap MnS in the type-I structure is the potential barrier both for electrons and holes, which means only the photogenerated carriers in MnS can pass the heterointerface. Thus, the photoresponse of this type-I heterojunction depends on the wide-bandgap material MnS. As the wavelength increases, photon energy is less than the energy required to pass through the MnS band gap; accordingly, the contribution of MnS to the photoresponse gradually decreases, resulting in a gradually smaller photocurrent. In contrast, the optical response characteristics of the device under reverse bias are different, which illustrates that the band alignment and carrier transport process of the type-I MnS/WSe_2_ heterojunction is tunable under electric field modulation.

## 4. Conclusions

In summary, WSe_2_ prepared by a mechanical tape exfoliation method and non-layered MnS grown by CVD are transferred to SiO_2_(300 nm)/Si substrate by a wet transfer process to constitute vertical structures, and an FET photodetector based on the MnS/WSe_2_ heterojunction is prepared by standard lithography process. The heterostructure is characterized by XPS, XRD, and HRTEM. The type-I band structure of the MnS/WSe_2_ heterojunction is confirmed by the first-principles calculations. The potential barrier height is regulated by the external electric field, which enables the efficient separation of photogenerated carriers. Consequently, the recombination of photogenerated carriers in the wideband system is inhibited due to the modulated band alignment, and the carrier collection efficiency is significantly improved, which is conducive to the realization of high-performance light detection. Finally, the heterojunction device exhibits a current on/off ratio over 10^3^ and an excellent photoresponsivity of 108 A W^−1^ in the visible range, and it can respond in UV–visible–near-infrared band with a fast response speed. The facilitation of carrier accumulation caused by the interface band bending on photoresponse performance has also been proved. In addition, the heterojunction can work in self-power mode and exhibits a photovoltaic effect. It is demonstrated that the 2D non-layered MnS/WSe_2_ type-I heterojunction exhibits promising application prospects in broadband response photodetectors and photovoltaic devices. This work is instructive for the rational design and modulation of type-I band structures to fabricate high-performance electronic and optoelectronic devices based on 2D non-layered materials.

## Data Availability

Data are contained within the article.

## Supplementary Materials

The following supporting information can be downloaded at: https://www.mdpi.com/article/10.3390/ma17071590/s1, Figure S1: (a) Optical micrograph of device using MnS nanosheets. (b) The I_ds_–V_ds_ curve of the MnS at V_gs_ = 0 V. (c) The transfer (I_ds_–V_gs_) curve of MnS at V_ds_ = 2 V. Figure S2: (a–d) Band alignments of MnS and WSe_2_ with 1~4 layers before contact. (e) The band offsets of CBM and VBM between MnS and WSe_2_ with 1~4 layers. Figure S3: Under the reverse bias, separation of electron–hole pairs in energy bands under the irradiation of 1550 nm laser. Figure S4: Noise spectral density as a function of frequency at V_ds_ = −2 V, V_gs_ = 0 V. Table S1: Properties of heterojunction MnS and WSe_2_. Table S2: The performance of broadband photodetector based on 2D heterojunctions. References [35,36,37,38,39] are cited in the Supplementary Materials.
