# Supplementary material for: A Broadband Photodetector Based on Non-Layered MnS/WSe2 Type-I Heterojunctions with Ultrahigh Photoresponsivity and Fast Photoresponse
Source: Materials (Basel). 2024 Mar 30;17(7):1590. doi: 10.3390/ma17071590 (PMC11012445; doi:10.3390/ma17071590)
Supplement: Supplementary file 1 [file materials-17-01590-s001.zip › materials-2902016-supplementary.pdf]

materials

Article

A Broadband Photodetector Based on Non-Layered MnS/WSe_2 Type-I Heterojunctions with Ultrahigh Photoresponsivity and Fast Photoresponse

Chaojie Xie †, Yibin Yang †, Kunle Li, Xuanhao Cao, Shanshan Chen * and Yu Zhao *

Guangdong Provincial Key Laboratory of Information Photonics Technology, Guangdong Provincial Key Laboratory of Functional Soft Condensed Matter, School of Material and Energy, Guangdong University of Technology, Guangzhou 510006, China; 2112102193@mail2.gdut.edu.cn (C.X.); yangyibin@gdut.edu.cn (Y.Y.); 1112302011@mail2.gdut.edu.cn (K.L.); cxh2903@gmail.com (X.C.)

* Correspondence: chensunny@gdut.edu.cn (S.C.); zhaoyu@gdut.edu.cn (Y.Z.)

† These authors contributed equally to this work.

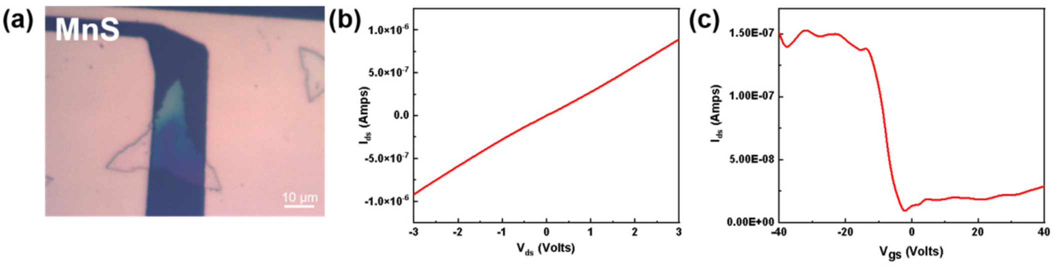

Figure S1. (**a**) Optical micrograph of device using MnS nanosheets. (**b**) The I_{ds}-V_{ds} curve of the MnS at V_g=0 V. (**c**) The transfer (I_{ds}-V_{gs}) curve of MnS at V_{ds}=2 V.

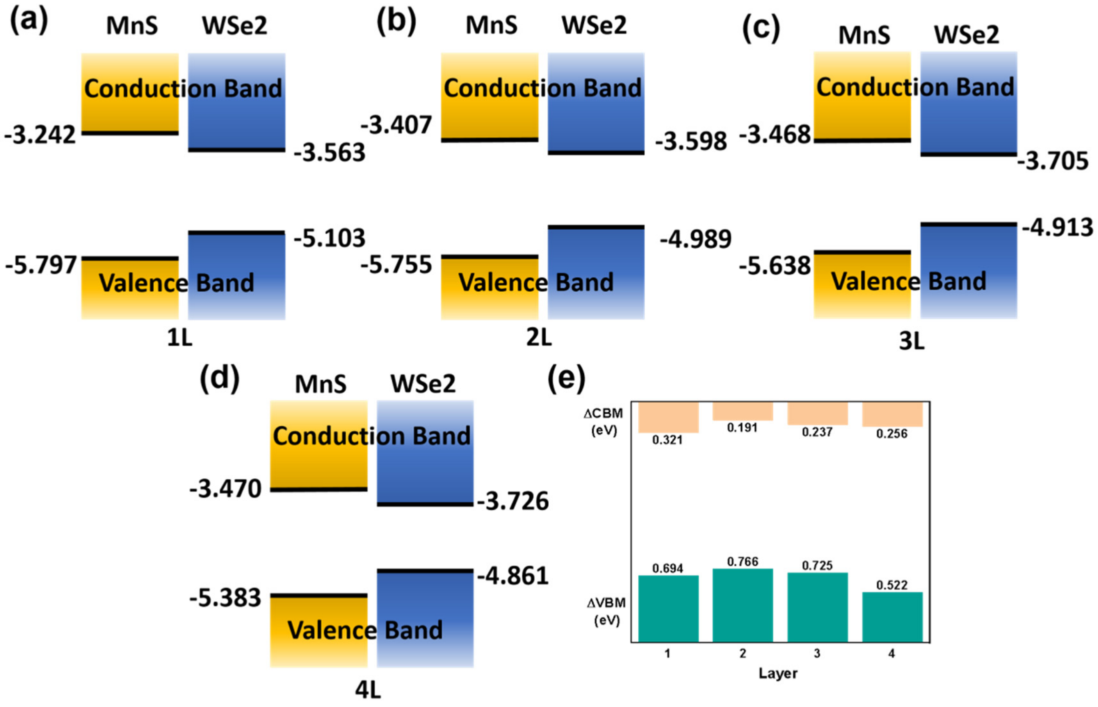

Figure S2. (**a**–**d**) Band alignments of MnS and WSe_2 with 1~4 layers before contact. (**e**) The band offsets of CBM and VBM between MnS and WSe_2 with 1~4 layers.

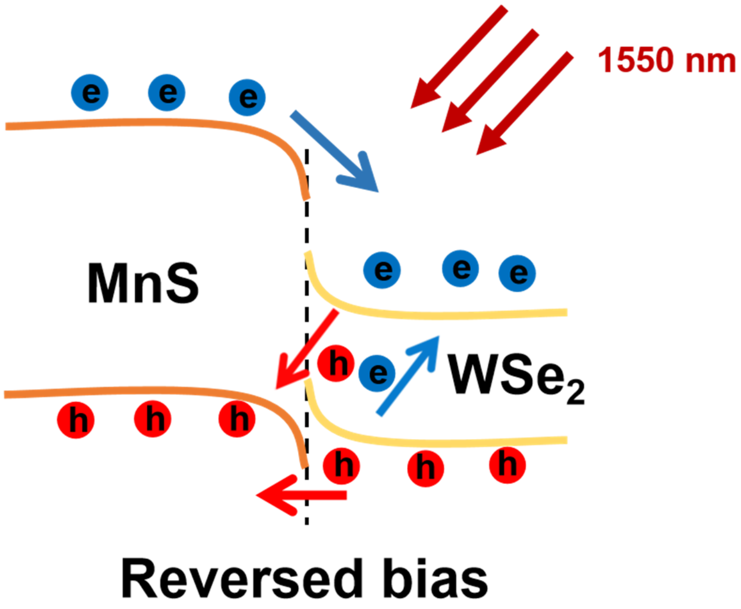

Figure S3. Under the reverse bias, separation of electron hole pairs in energy bands under the irradiation of 1550 nm laser.

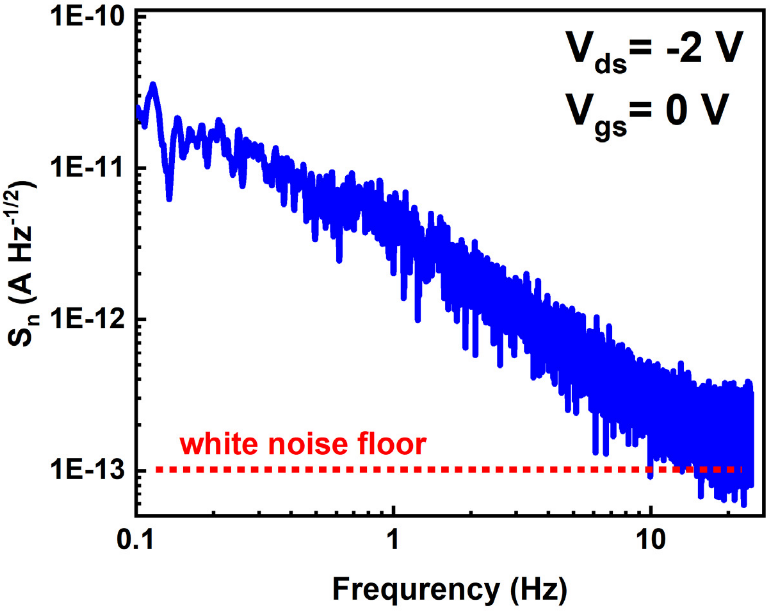

Figure S4. Noise spectral density as a function of frequency at V_{ds} = -2 V, V_{gs} = 0 V.

Table S1. Properties of heterojunction MnS and WSe_2.

Device	Wavelength [nm]	Responsivity [A/W]	Response time (up/down) [ms]
MnS/WSe_2	532	108	19/10
MnS	532	7.2	5000/4500
WSe_2	532	2.1	220/310

Table S2. The performance of broadband photodetector based on 2D heterojunctions.

Heterojunction	Response range [nm]	Responsivity [A/W]	Detectivity [jones]	rise/fall time[ms]	Reference
MnS/WSe_2	365-1550	108	3.5×10^{12}	19/10	This work
WS_2/Si	400-1100	1.1	5×10^{11}	42/76	[1]
$MoS_2/BP/Si$	532-1550	22.3	3.1×10^{11}	0.015	[2]
WS_2/Bi_2Te_3	370-1550	30.4	2.3×10^{11}	20/20	[3]
β-In_2Se_3/Si	265-1300	6.4	4.3×10^{10}	0.002/0.004	[4]
MoS_2 /Ge	406-1550	3	~	10	[5]

1. Chowdhury, R.K.; Maiti, R.; Ghorai, A.; Midya, A.; Ray, S.K. Novel silicon compatible p-WS_2 2D/3D heterojunction devices exhibiting broadband photoresponse and superior detectivity. *Nanoscale* **2016**, *8*, 13429–13436.
2. Ye, L.; Li, H.; Chen, Z.F.; Xu, J. Near-Infrared Photodetector Based on MoS_2/Black Phosphorus Heterojunction. *ACS Photonics* **2016**, *3*, 692–699.
3. Yao, J.; Zheng, Z.; Yang, G. Layered-material WS_2/topological insulator Bi_2Te_3 heterostructure photodetector with ultrahigh responsivity in the range from 370 to 1550 nm. *J. Mater. Chem. C* **2016**, *4*, 7831–7840.
4. Guo, H.; Xia, Y.; Yu, Y.; Zhou, R.; Niu, H.; Mao, X.; Wan, L.; Xu, J. High-speed and broadband spectral photodetectors based on beta-In_2Se_3/Si het-erojunction. *Mater. Sci. Semicond. Process.* **2022**, *138*, 106304.
5. Hwang, A.; Park, M.; Park, Y.; Shim, Y.; Youn, S.; Lee, C.H.; Jeong, H.B.; Jeong, H.Y.; Chang, J.; Lee, K.; et al. Visible and infrared dual-band imaging via Ge/MoS_2 van der Waals heterostructure. *Sci. Adv.* **2021**, *7*, eabj2521.
